# Different patterns of failure in two treatment regimens for primary central nervous system lymphoma, a retrospective analysis of 124 cases in Taiwan

**DOI:** 10.1007/s10238-023-01182-2

**Published:** 2023-09-07

**Authors:** Chin-Hsuan Chuang, Ming-Chung Kuo, Hung Chang, Jin-Hou Wu, Yu-Shin Hung, Che-Wei Ou, Tung-Liang Lin, Yi-Jiun Su, Yuen-Chin Ong, Lee-Yung Shih, Hsiao-Wen Kao

**Affiliations:** 1 Division of Hematology-Oncology, Department of Internal Medicine, Chang Gung Memorial Hospital at Linkou, No. 5, Fuxing Street, Guishan Dist., Taoyuan City, 333423 Taiwan, ROC; 2 College of Medicine, Chang Gung University, Taoyuan, Taiwan

**Keywords:** Primary CNS lymphoma, IELSG score, MSKCC score, MATRix regimen, High dose methotrexate

## Abstract

To explore prognostic factors and outcomes of primary central nervous system lymphoma (PCNSL) of diffuse large B-cell lymphoma (DLBCL) in Taiwan, 124 PCNSL-DLBCL patients (from 1995 to 2021) were retrospectively analyzed. Mainly, two treatment modalities including sandwich chemoradiotherapy and modified MATRix regimen were employed in these patients. Overall survival (OS) was determined by log-rank test and time-dependent Cox analysis. Median OS of all patients was 27.1 months. 47 (37.9%) patients who underwent sandwich chemoradiotherapy had a complete remission (CR) rate of 87.2%, median OS of 53.9 months, and progression free survival (PFS) of 42.9 months. 11 (8.9%) patients who underwent modified MATRix regimen had CR rate of 72.7%, median OS of 18.9, and PFS of 11.2 months. There are no significant OS differences between treatment groups or addition of Rituximab. Patients treated with the modified MATRix regimen experienced a higher early mortality rate followed by a survival plateau. IELSG low-risk group had significantly improved OS and PFS than IELSG intermediate- or high-risk group. In multivariant analysis, age > 60 years old and bilateral cerebral lesions are associated with significantly inferior OS. Sandwich chemoradiotherapy demonstrated better early survival and reduced treatment-related toxicity for PCNSL patients compared to the modified MATRix regimen. However, the long-term follow-up revealed a higher rate of treatment failure events in the sandwich chemoradiotherapy group. IELSG and MSKCC scores served as reliable risk assessment models. Incorporating bilateral cerebral lesions as a risk factor further improved risk evaluation.

**Supplementary Information:**

The online version contains supplementary material available at 10.1007/s10238-023-01182-2.

## Introduction

Primary diffuse large B-cell lymphoma (DLBCL) of the central nervous system (PCNSL) is a recognized subtype of non-Hodgkin lymphoma by the 2017 WHO classification, and 2022 International Consensus classification, and is revised as primary large B-cell lymphoma of immune-privileged sites by the 2022 WHO classification [1–3]. PCNSL is characterized by its exclusive origin in the central nervous system (CNS), which includes the brain, spinal cord, and eyes. Several factors contribute to its development, including a weakened immune system, exposure to immunosuppressive drugs, and viral infections such as Epstein-Barr virus (EBV). People with human immunodeficiency virus (HIV) infection or other immunocompromised conditions are at higher risk of developing PCNSL [4]. While both PCNSL and systemic B cell lymphomas arise from B cell lineage [5], PCNSL has been debated to correspond to either the activated B cell-like or germinal center B cell-like subtype. PCNSL displays characteristics of mature B cells and shares similarities with late germinal center B cells. Notably, PCNSL shows a higher prevalence of CD79B and MYD88 mutations compared to systemic DLBCL [5, 6].

Treatment of PCNSL has evolved over the last decade. Experts agree that high-dose methotrexate (HD-MTX) is the backbone of multimodal therapy [4]. After the implementation of HD-MTX-based chemotherapy, accompanied by consolidating radiotherapy, the overall response rate remained high, ranging from 71 to 94%. The median overall survival (OS) was reported to be between 30 and 60 months, with five-year OS rates ranging from 30 to 50% [7]. However, patients treated with chemoradiation developed neurotoxicity [8]. It was also found that deferring radiotherapy did not adversely impact the OS in older patients [8, 9]. Despite the high sensitivity to conventional chemotherapy and radiotherapy, remissions are frequently short lasting [10]. Previous clinical trials of PCNSL usually excluded patients with older age or poor performance status, and may not be suitable for the typical PCNLS patient population encountered in routine clinical practice [7, 11, 12].

The International Extranodal Lymphoma Study Group (IELSG) score system is a widely adopted tool for predicting the outcome of PCNSL patients [2, 3]. The score is determined based on several factors including age, performance status, tumor location, cerebrospinal fluid (CSF) protein level, and serum lactate dehydrogenase (LDH) level [13]. Another prognostic model, Memorial Sloan-Kettering Cancer Center (MSKCC) score based on age and performance status, is also used to predict outcomes for newly diagnosed PCNSL patients [14].

This study aimed to determine the clinical features, outcomes based on different treatment protocols, and prognostic factors for PCNSL patients in Taiwan with the goal of improving future clinical practices.

## Patient and methods

### Patient selection

Between 1995 and 2021, a total number of 124 patients aged above 18 years old with newly diagnosed PCNSL of DLBCL type at Chang Gung Memorial Hospital, Linkou branch were enrolled. The diagnosis of PCNSL was based on criteria established by the 2017 WHO classification [1]. The study was conducted through a retrospective review of medical records and approved by the Investigational Review Board of Chang Gung Memorial Hospital (202100653B0).

The initial evaluation consisted of a complete medical history, physical examination, and laboratory tests. Brain imaging was conducted using computerized tomography (CT) scan or Magnetic Resonance Imaging (MRI). CT scan of the brain, chest, abdomen, and pelvis or Positron Emission Tomography (PET/CT) is evaluated to exclude non-CNS involvement of lymphoma. The Eastern Cooperative Oncology Group (ECOG) scale was used to assess performance status (ranging from 0 to 4). The risk stratification was assigned based on the IELSG and MSKCC scores [13, 14]. The last data cut-off date for follow-up is September 29, 2022.

### Treatment protocol

The study primarily used two treatment modalities. Protocol S, adopted from the combined modality and started in 2008, consisted of sandwich pre-radiotherapy systemic intermediate dose of MTX (ID-MTX) 1 g/m^2^ plus six doses of intrathecal methotrexate at 12 mg per dose, cranial radiotherapy (4000 cGy whole-brain radiotherapy [WBRT] plus a 1440 cGy boost), and two cycles of high-dose Cytarabine, with each course consisting of two doses of 3 g/m^2^ Cytarabine separated by 24 h [15].

Protocol M, modified from MATRix protocol and started in 2018, involved the combination chemotherapy with HD-MTX (3.5 g/m^2^) and Cytarabine (2 g/m^2^) plus Rituximab and followed by WBRT, with dose adjustments according to age and response [11]. Thiotepa was omitted from the modified protocol M as it was not available in Taiwan. Intrathecal methotrexate was given if lymphoma was present in the CSF at diagnosis. Other treatment options included ID-MTX (1 g/m^2^) combined with WBRT or WBRT alone.

### Treatment response evaluation

The treatment response was evaluated by CT scans of the whole body including the brain, or MRI of the brain, which were performed after the completion of therapy and every 3 to 6 months for the first two years post-therapy. The response to treatment was determined based on the revised response criteria for malignant lymphoma and categorized as complete remission (CR), partial response (PR), stable disease (SD), or progressive disease (PD) [16].

### Statistical analysis

Wilcoxon’s rank-sum test, Fisher’s exact test, and the χ^2^ analysis were used whenever appropriate to compare groups. Progression-free survival (PFS) was defined as the time between the date of treatment and the date of progression, relapse, or death. OS was defined as the time between biopsy and death. Survival estimates were calculated by the Kaplan–Meier method and compared by Log-rank test. Results were expressed as hazard ratios (HR) with 95% confidence intervals (CI) for progression or death in univariate analyses, and factors with *P* values less than 0.05 in univariate analyses were included in multivariate analyses by Time-dependent Cox regression model. In all analyses, two-sided *P* values lower than 0.05 were statistically significant. All statistical analysis was performed with R version 4.2.1, R Foundation for Statistical Computing, Vienna, Austria.

## Results

### Patient characteristics

The clinical characteristics of 124 PCNSL-DLBCL patients are listed in Table 1. The median age of diagnosis was 63 years (range 18–84), with 50.0% being male. 70.9% (88/124) of patients had an ECOG performance status of two or higher at diagnosis. Only 3.7% (4/107) of patients were infected with HIV. The most common symptom at presentation was motor impairment (49.1%). The frequency of IELSG scores for low, intermediate, and high-risk groups was 13.7%, 37.1%, and 31.5%, respectively. In terms of the MSKCC classification, 19.4% were categorized as Class I, 21.8% as Class II, and 58.9% as Class III. The median OS was 27.1 months (95% CI 19.9–36.9) and the median PFS was 19.1 months (95% CI 12.1–31.6) for all 124 patients.

**Table 1:** Clinical characteristics of 124 patients with PCNSL

Characteristic	No. of patients (N = 124)	%
Age (years)		
Median (range)	63 (18–84)	
> 60 years	75	60.4
Male gender	62	50.0
ECOG (PS)		
0–1	36	29.0
≥ 2	88	70.9
EBV positive (EBER, EBV DNA)	9 (97 unknown)	7.2
HIV positive (17 unknown)	4/107	3.7
Clinical symptoms		
Motor	61	49.1
Sensory (visual)	16	12.9
Consciousness change	17	13.7
Cognitive, personality change	5	4.0
IICP symptoms and signs	9	7.2
Seizure	9	7.2
Characteristic (N = 124)	n/N	%
LDH > 1 x	35/115	30.4
High CSF protein level	54/84	64.2
Positive CSF cytology	11/116	9.5
Bilateral cerebellar lesions	51/124	41.1
Deep structure involvement	75/124	60.5
Prognostic group by IELSG score		
Low (0–1)	17	13.7
Intermediate (2–3)	46	37.1
High (4–5)	39	31.5
Unknown	22	17.7
Prognostic group by MSKCC class		
I	24	19.4
II	27	21.8
III	73	58.9
Diagnostic methods		
Stereotactic biopsy	74	59.7
Surgical resection	48	38.7
Aspiration	1	0.8
Treatment		
Protocol S	47	37.9
Protocol M	11	8.9
ID-MTX + RT	20	16.1
RT alone	26	21.0
Other	14	11.3
Loss follow up	6	4.8

*PS* Performance status; *EBER* EBV-encoded small RNAs; *IICP* Increased intracranial pressure; *LDH* Lactate dehydrogenase; *CSF* Cerebrospinal fluid; *IELSG* International extranodal lymphoma study group; *MSKCC* Memorial sloan-kettering cancer center; *ID-MTX* Intermediate-dose methotrexate; *RT* Radiotherapy; *HIV* Human immunodeficiency virus

### Treatment response and outcome of PCNSL patients based on treatment protocol

Among 58 (46.8%) patients who received aggressive systemic therapy, 47 (37.9%) underwent the sandwich combined modality (Protocol S) while 11 (8.9%) underwent the modified MATRix regimen (Protocol M). 20 patients (16.1%) received ID-MTX with WBRT, while 26 (21.0%) received WBRT only. Another 14 patients (11.3%) only received palliative care or could not complete their treatment, and six patients (4.8%) were lost to follow-up. The choice of treatment was based on the year of diagnosis, physicians’ decision, patients’ performance status and preference.

**Fig. 1:**
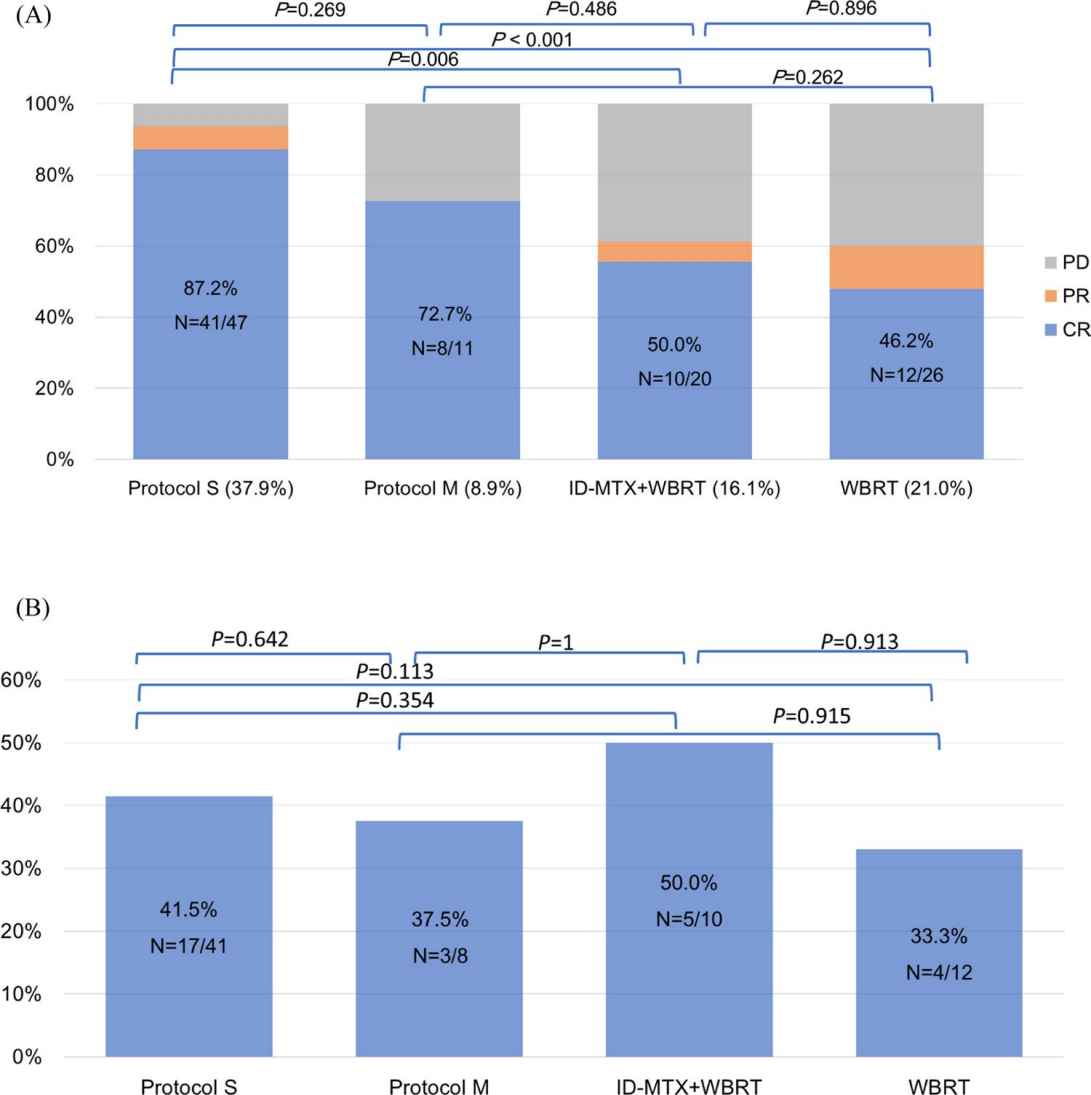
Treatment response. **A** Response rate according to treatment protocols, classified as CR (complete response), PR (partial response), and PD (progressive disease). **B** Relapse rate of CR patients according to treatment protocols

**Fig. 2:**
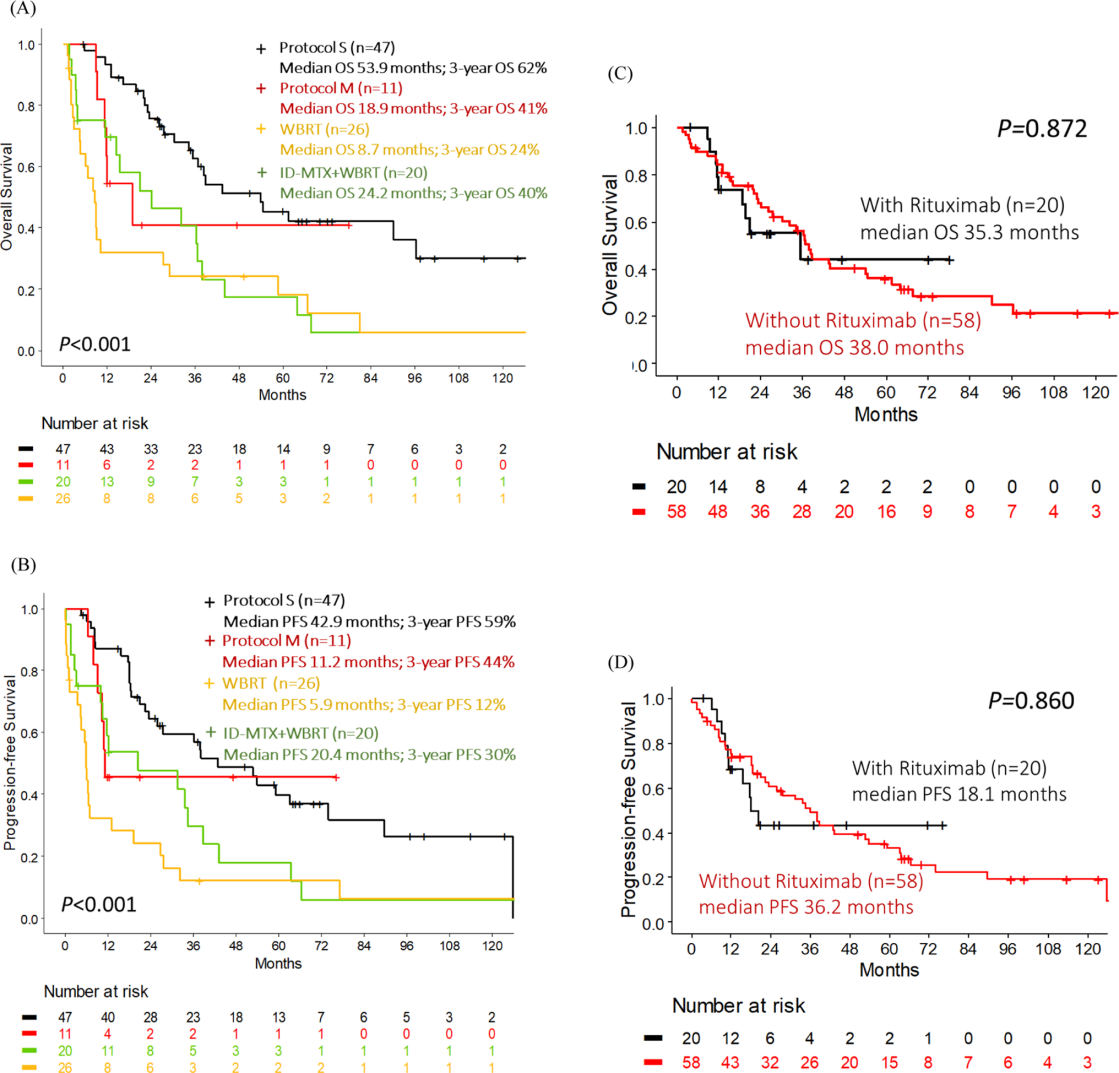
Outcomes according to treatment protocols. **A** Overall survival according to treatment protocols. **B** Progression-free survival according to treatment protocols. **C** Overall survival by adding Rituximab or not in patients treated with intensive chemotherapy. **D** Progression-free survival by adding Rituximab or not in patients treated with intensive chemotherapy

There is no significant difference in the clinical characteristics between patients treated with protocol S and protocol M (Supplementary Table 1). Patients who underwent Protocol S had a CR rate of 87.2% (Fig. 1A), a relapse rate of 41.5% (Fig. 1B), a median OS of 53.9 months (95% CI 35.3-not reached [NR], Fig. 2A), and a median PFS of 42.9 months (95% CI 25.9–89.7, Fig. 2B). Patients who underwent Protocol M (modified MATRix regimen) had a CR rate of 72.7%, a relapse rate of 37.5%, a median OS of 18.9 months (95% CI 11.7-NR), and a median PFS of 11.2 months (95% CI 10.1-NR). Patients who received ID-MTX (1 g/m^2^) plus WBRT had a CR rate of 50.0%, a relapse rate of 50.0%, a median OS of 24.2 months (95% CI 14.5–44.0, Fig. 2A), and a median PFS of 20.4 months (95% CI 10.5–43.2). Those who received WBRT only had a CR rate of 46.2%, a relapse rate of 33.3% (Fig. 1A, B), a median OS of 8.7 months (95% CI 4.8–29.0), and a median PFS of 5.9 months (95% CI 4.3–19.3).

Patients who received the sandwich strategy with Protocol S had significantly improved OS compared to patients who received ID-MTX plus WBRT (*P* = 0.048) and WBRT alone (*P* < 0.001). However, there was no difference between the sandwich Protocol S and Protocol M with modified MATRix regimen (*P* = 0.25). Early failure followed by survival curve plateau for PFS and OS was observed in Protocol M with modified MATRix regimen, but not in Protocol S (Fig. 2A, B).

The overall treatment-related mortality rate is 8.7% (9/104), with 4.3% (2/47) among patients who received protocol S, and significantly higher in 36.4% (4/11) for those under protocol M (*P* = 0.006). Among the 11 patients treated with Protocol M, four experienced therapy-related mortality during the early phase, with all deaths attributed to severe infections. (Supplementary Table 1).

### The impact of adding Rituximab on the outcome of PCNSL patients

The outcome of those who received high-dose chemotherapy with or without Rituximab was analyzed (Fig. 2C, D). Patients who received chemotherapy with Rituximab had a CR rate of 70.0%, a relapse rate of 33.3%, a median OS of 35.3 months (95% CI 18.9-NR), and a median PFS of 18.1 months (95% CI 15.6-NR). Patients who did not receive Rituximab had a CR rate of 75.9%, a relapse rate of 45.4%, a median OS of 38.0 months (95% CI 30.4–61.5), and a median PFS of 36.2 months (96% CI 23.5–59.1). There were no significant differences in the CR rate, relapse rate, PFS, and OS between patients who received high-dose chemotherapy with or without Rituximab. However, PFS and OS plateau seemed to be observed earlier in patients with Rituximab than in patients without Rituximab.

Further analysis was performed on patients who underwent Protocol S with or without Rituximab (Supplementary Fig. 1). The median OS and PFS were not reached yet in seven patients who received Protocol S with Rituximab. On the other hand, 40 patients who received Protocol S without Rituximab had a median OS of 54.0 months (95% CI 34.1-NR) and a median PFS of 43.0 months (95% CI 25.9–89.7). Adding Rituximab to chemotherapy seemed to improve the outcome but without significant statistical differences in terms of OS and PFS.

### Risk factors of PCNSL patients

**Fig. 3:**
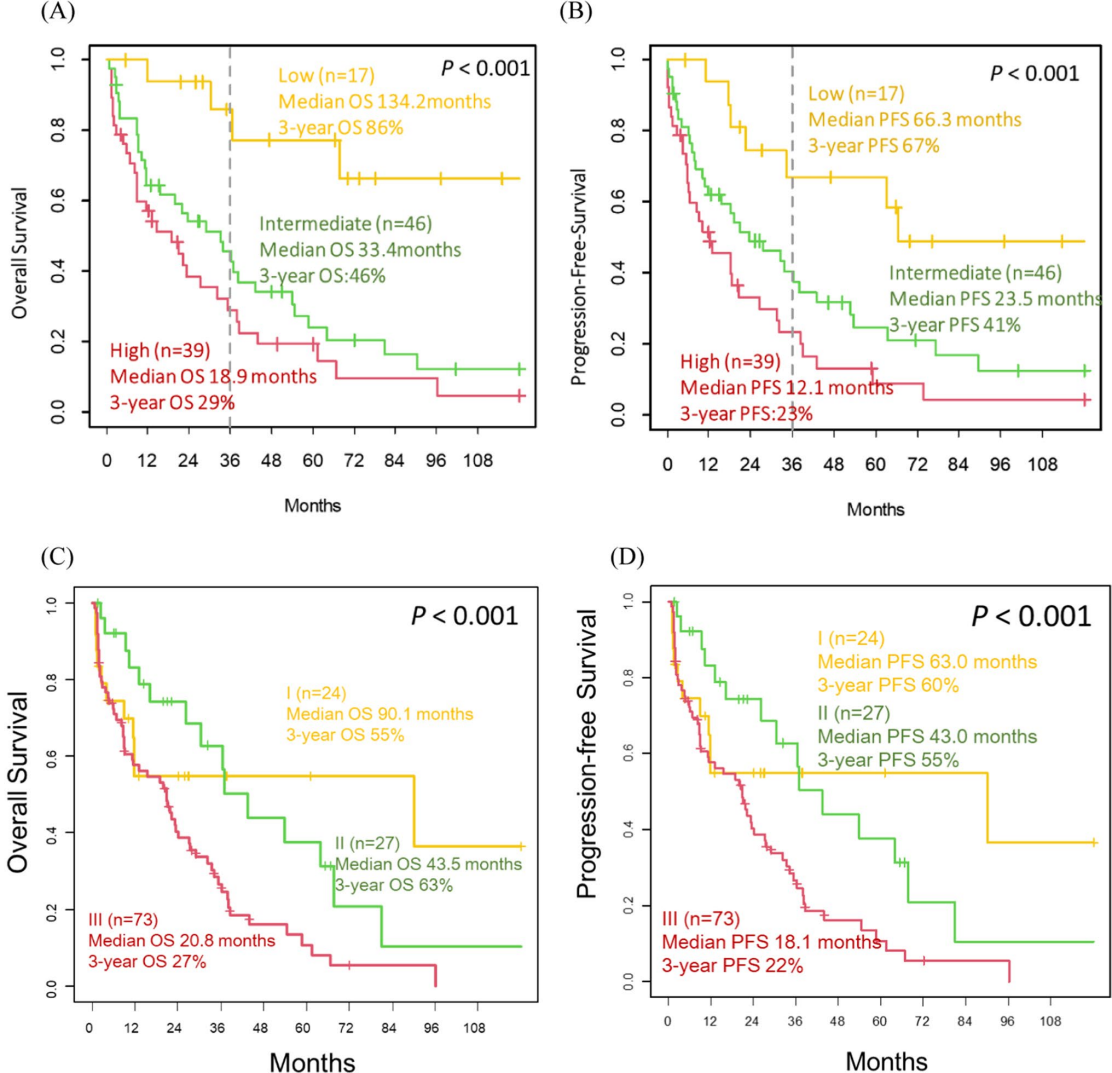
Outcome based on risk stratification. **A** Overall survival according to IELSG group. **B** Progression-free survival according to IELSG group. **C** Overall survival according to MSKCC class. **D** Progression-free survival according to MSKCC class

Patients in the low, intermediate, and high-risk groups according to IELSG score had median OS of 134.2 (95% CI 67.6-NR), 33.4 (95% CI 15.6–54.6), and 18.9 (95% CI 8.9–35.3) months, as well as median PFS of 66.3 (95% CI 64.4-NR), 23.5 (95% CI 11.8-NR), and 12.1 (95% CI 6.1–26.8) months, respectively (Fig. 3A, B). Patients in the low-risk group had significantly better OS and PFS compared to those in the intermediate or high-risk groups (*P* < 0.001). No significant difference was found between the intermediate-risk group and high-risk group in terms of OS and PFS. Regarding the MSKCC score, patients in the class I, class II, and class III had median OS of 90.1 (95% CI 11.4-NR), 43.5 (95% CI 30.4-NR), and 20.8 (95% CI 11.3–27.5) months, as well as median PFS of 63.0 (95% CI 11.2-NR), 43.0 (95% CI 22.5-NR), and 18.1 (95% CI 7.8–20.9) months, respectively (Fig. 3C, D). Patients in class III had significantly inferior OS and PFS compared to those in class I or class II (*P* < 0.001). No significant difference was found between class I and class II in terms of OS and PFS.

After excluding 20 patients who did not receive standard treatment or were lost to follow-up, a total of 104 patients were included in the Cox regression model to identify risk factors for OS (Table 2). Univariate analysis revealed inferior OS is significantly associated with age over 60 years (HR 2.0, 95% CI 1.2–3.4, *P* = 0.009), ECOG ≥ 2 (HR 1.8, 95% CI 1.1–3.1, *P* = 0.025), and the presence of bilateral cerebral lesions (HR 1.8, 95% CI 1.1–2.9, *P* = 0.02), deep lesion (HR 1.7, 95% CI 1.0–2.8, *P* = 0.039). Multivariate analysis showed age over 60 years (HR 2.1, 95% CI 1.2–3.6, *P* = 0.01) and bilateral cerebral lesions (HR 1.8, 95% CI 1.0–3.2, *P* = 0.04) to be independent adverse factors for OS with variables including age, ECOG, deep lesions, and bilateral cerebral lesions.

**Table 2:** Univariate and multivariate overall survival analysis

	Univariate analysis		Multivariate analysis	
Variable	HR (95% CI)	*P* value	HR (95% CI)	*P* value
Age > 60 years old	2.01 (1.19–3.40)	0.009*	2.07 (1.18–3.62)	0.01*
Male sex	1.07 (0.67–1.71)	0.776		
ECOG ≥ 2	1.83 (1.08–3.11)	0.025*	1.23 (0.67–2.24)	0.51
Bilateral cerebral lesions	1.79 (1.09–2.94)	0.020*	1.80 (1.02–3.18)	0.04*
Cerebellar lesion	1.74 (0.98–3.09)	0.061		
Deep lesion	1.70 (1.03–2.82)	0.039*	1.16 (0.64–2.12)	0.62
CSF protein	1.09 (0.59–2.02)	0.783		
Elevated LDH	0.78 (0.44–1.36)	0.375		
HIV positive	0.29 (0.04–2.11)	0.113		

*HR* Hazard ratio; *CI* Confidence interval; *LDH* Lactate dehydrogenase; *ECOG* Eastern cooperative oncology group; *HIV* Human immunodeficiency virus; *CSF* Cerebrospinal fluid

*Statistical significance with *P* values less than 0.05

## Discussion

Our study of 124 patients with DLBCL-type PCNSL provided real-world experience on clinical characteristics, treatment response, and outcomes. This is the largest PCNSL series reported in Taiwan [17]. Patients were mainly older adults (median age 63) with poor ECOG performance status (70.9% ECOG ≥ 2) and a median OS of 27.1 months. There is no difference in CR rates, relapse rates, PFS, and OS between patients treated with sandwich protocol S and patients treated with protocol M modified from the MATRix regimen. Patients who received protocol S had significantly higher CR rate, as well as better PFS and OS compared to patients treated with ID-MTX plus WBRT or WBRT alone. Adding Rituximab did not significantly improve PFS and OS, but showed an earlier survival plateau. IELSG and MSKCC scores are both useful for risk stratification in PCNSL patients from this cohort. Age of more than 60 years old and bilateral cerebral lesions were independent prognostic factors for PCNSL patients in this study. Our findings on the clinical features of patients with DLBCL-type PCNSL align with previous reports, including a trend towards older age and occurrence in immunocompetent individuals [17, 18]. We compared the results with that of the IELSG 32 trial and found that patients in Taiwan with DLBCL-type PCNSL had a higher average age (63 vs. 57 years old) and a worse performance status (ECOG ≥ 2:70% vs. 33%) [11].

EBV-associated lymphoma accounts for a varying percentage of PCNSL ranging from 4.8% to 13.6% in different studies [15–17]. In our study, 7.2% (9/124) of patients were EBV positive (detected by EBV-encoded small RNAs or serum EBV DNA), but due to a lack of testing in most patients (97 unknown), the data is less reliable. EBV-associated PCNSL in immunosuppressed patients is immunobiologically distinct from EBV-negative and HIV-negative PCNSL [19]. Restoration of EBV-specific T-cell immunity has been shown to induce a clinical response in EBV-positive lymphomas [19, 20]. Hence, the role of EBV in the development of PCNSL warrants further investigation in clinical practice.

HIV-associated PCNSL was reported in 6.1% of PCNSL patients and varied by geographical location [21]. Our study found a 3.7% HIV rate. Before the introduction of antiretroviral therapy (ART), HIV-associated PCNSL OS was less than two months. Although OS has improved with ART-associated immune reconstitution, the median OS is generally reported to be less than one year [22]. In our study, four HIV-positive patients enrolled were in the Acquired Immunodeficiency Syndrome (AIDS) stage. The median OS of HIV patients was not reached (95% CI 0.9-NR), compared to 27.5 months (95% CI 20.8–36.9) for HIV-negative patients. Notably, two patients who completed Protocol S in addition to ART therapy experienced long-term survival benefits, and another one patient died early due to sepsis during the treatment. Therefore, AIDS patients receiving chemotherapy should be vigilant about the risk of early mortality due to severe infections resulting from immunocompromised status.

Several risk scores are available for the risk stratification of PCNSL patients. The IELSG score, consisting of age, performance status, serum LDH level, CSF protein level, and tumor location, is widely used for risk stratification in PCNSL [13]. The MSKCC also proposed a prognostic model solely based on age and performance status in 2006 [14, 23]. We found that age greater than 60 years old, an ECOG performance status greater than 1, deep lesion and bilateral cerebral lesions were adverse factors for OS in univariate analysis. By multivariate analysis, age and bilateral cerebral lesions were independent risk factors for OS. As a result, it may be worth considering incorporating tumor site as a prognostic factor in future studies.

In our study, we observed a substantial overall response rate to standard frontline therapy, with a CR rate ranging from 72.1% to 87.2%. However, patients who underwent high-dose chemotherapy still had a high rate of relapse (37.5–41.5%) in our study. In contrast, the PRECIS study reported lower relapse rates of 30.3% and 4.5% after intensive chemotherapy with consolidation using WBRT and autologous stem cell transplantation (ASCT), respectively [8]. In the IELSG 32 trial, the MATRix chemoimmunotherapy regimen resulted in an overall response rate of 79% and a 7-year OS rate of 56%. However, when patients were treated with the MATRix regimen and underwent consolidation therapy, the 7-year OS rate improved to 70%, and relapse rate of 20–25% without difference between WBRT and ASCT [11, 24]. Consolidation therapy is critical to conquer the high relapse rate of PCNSL. The ILESG 32 trial utilized a treatment protocol consisting of induction therapy followed by consolidation therapy, which could either be ASCT or WBRT [11, 24]. The PRECIS study revealed that ASCT consolidation has a superior outcome and less neurotoxicity than WBRT in patients aged 60 years or younger [9, 25]. However, none of the patients enrolled in our study underwent ASCT as frontline consolidation therapy, which may have contributed to the high relapse rate. For patients who are ineligible for ASCT, WBRT remains an effective consolidation treatment. To minimize neurotoxicity, some studies suggest a practical approach of administering low-dose WBRT with higher doses targeted specifically to the tumor [26]. Further investigation is needed to determine the optimal radiotherapy strategy for transplant-ineligible PCNSL patients.

Patients receiving protocol S had fewer early adverse effects but continued to relapse during long-term follow-up. Of 11 patients who received Protocol M, 4 patients experienced therapy-related mortality in the early phase, all death due to severe infection. However, patients who completed protocol M treatment appeared to have long-term PFS and OS despite lack of ASCT.

Compared to the MATRix trial [11], our study included older patients with worse performance status, which are significant prognostic factors [13, 23]. In the elderly, some studies highlight that the first cycle of the MATRix regimen had severe toxicity, with 6% of patients requiring intensive care unit admission due to life-threatening infections, and infectious toxicities were reported in 11%–28% of each MATRix cycle [27, 28]. One pilot trial in Germany showed a promising outcome of CR rate, OS, and PFS among elderly (> 65 years old) patients by adopting a de-escalating treatment approach that reduced induction chemotherapy to two cycles, followed by ASCT immediately [29]. The ongoing OptiMATe trial aims to optimize induction treatment comprising only two cycles of the MATRix regimen after a pre-phase Rituximab/HD-MTX compared to the standard four cycles of MATRix followed by ASCT in considering high therapy-related mortality [28]. Recent studies have illuminated DLBCL and PCNSL pathobiology. DLBCL features frequent chromatin gene mutations (*EP300, CREBBP, KMT2D, SUZ12, EZH2*), suggesting potential disease-halting chromatin interventions [5, 6]. Microenvironment elements impact PCNSL [6]. Elevated VEGF connects to aggressive DLBCL and adverse prognosis [6]. Unveiling PCNSL's molecular pathways opens doors for innovative treatments, including epigenetic modifications, microenvironment interactions, and genetic target.

Our study has limitations. The single-hospital inclusion could introduce selection bias and limit generalizability. Retrospective data collection may lead to incomplete information and affect accuracy. The extended study duration could confound results due to changing treatment practices. Lack of randomization and not accounting for certain confounding factors add complexity. Subgroup analyses based on patient characteristics or treatments were limited. Genetic profiling and molecular subtype data are unavailable due to tool limitations in our constitution. The limitations of this study could be potentially solved through collaboration research networks as prospective multi-center studies or randomized clinical trials for more detailed data collection including genetic factors, consensus treatment modalities, and external validation of major findings.

## Conclusions

In conclusion, the patients with PCNSL were found to have an older age and a poorer performance status at the time of diagnosis in Taiwan. The IELSG and MSKCC scores are reliable risk assessment models for PCNSL. Factors identified as independent prognostic indicators include age more than 60 years old and bilateral cerebral involvement of tumors. Both the sandwich chemo-radiotherapy regimen and modified MATRix regimen appear to be viable options for treatment of PCNSL. However, there are trade-offs to take into account for intensified induction chemotherapy, including the balance between the risk of early mortality due to treatment toxicities and the benefit of long-term effectiveness. While the modified MATRix regimen may provide a durable response, the high risk of early treatment-related mortality is a concern, especially for older patients or patients with poor performance status. A de-escalating approach may be worth considering. Additionally, frontline consolidation with ASCT should be considered more frequently in the management of PCNSL. This approach has shown promise in improving long-term outcomes for PCNSL patients and should be further explored in randomized clinical trials.

### Supplementary Information

Below is the link to the electronic supplementary material.

Supplementary file1 (PDF 411 KB)

## Data Availability

The datasets generated during and/or analyzed during the current study are available from the corresponding author on reasonable request.
