# Supplementary material for: Different patterns of failure in two treatment regimens for primary central nervous system lymphoma, a retrospective analysis of 124 cases in Taiwan
Source: Clin Exp Med. 2023 Sep 7;23(8):5327–36. doi: 10.1007/s10238-023-01182-2 (PMC10725386; doi:10.1007/s10238-023-01182-2)

Article title: Different patterns of failure in two treatment regimens for primary central nervous system lymphoma, a retrospective analysis of 124 cases in Taiwan

Journal name: Clinical and Experimental Medicine

Author names: Chin-Hsuan Chuang, Ming-Chung Kuo, Hung Chang, Jin-Hou Wu, Yu-Shin Hung, Che-Wei Ou, Tung-Liang Lin, Yi-Jiun Su, Yuen-Chin Ong, Lee-Yung Shih, Hsiao-Wen Kao

Corresponding author: Hsiao-Wen Kao, Division of Hematology-Oncology, Department of Internal Medicine, Chang Gung Memorial Hospital at Linkou, No.5, Fuxing Street, Guishan Dist., Taoyuan City 333423, Taiwan (R.O.C.). E-mail: hsiaowen@cgmh.org.tw. Tel: +886-3-3281200#2524. Fax: 886-3-3286697.

**Supplementary Table 1. Clinical characteristics of 124 patients according to treatment protocols**

| Characteristic | All patients (N=124) | Protocol S (N=47) | Protocol M (N=11) | *P* (S vs. M) | ID-MTX+RT (N=20) | RT alone (N =26) | *P* (All) |
|---|---|---|---|---|---|---|---|
| **Age (years)** | | | | | | | |
| Median (Range) | 63 (18-84) | 61 (25-82) | 55 (26-68) | 0.127 | 66 (48-83) | 62 (23-84) | <0.001 |
| > 60 years | 75 (60.4) | 25 (53.2) | 3 (27.3) | 0.225 | 16 (80.0) | 23 (88.5) | <0.001 |
| **Male Gender** | 62 (50.0) | 24 (51.1) | 6 (54.5) | 1 | 10 (50.0) | 11 (42.3) | 0.854 |
| **ECOG** | | | | 0.799 | | | <0.001 |
| 0-1 | 36 (29.0) | 35 (74.5) | 5 (45.5) | | 5 (25.0) | 3 (11.5) | |
| ≥ 2 | 88 (70.9) | 12 (25.5) | 6 (54.5) | | 15 (75.0) | 23 (88.5) | |
| **Prognostic group by IELSG score** | | | | 0.762 | | | 0.002 |

| | | | | | | | |
|---|---|---|---|---|---|---|---|
| Low (0-1) | 17 (13.7) | 11 (23.4) | 4 (36.3) | | 2 (10.0) | 0 | |
| Intermediate (2-3) | 46 (37.1) | 17 (36.2) | 5 (45.5) | | 7 (35.0) | 9 (34.6) | |
| High (4-5) | 39 (31.5) | 12 (25.5) | 2 (18.2) | | 9 (45.0) | 11(42.3) | |
| Unknown | 22 (17.7) | 7 (14.9) | 0 | | 2 (10.0) | 6 (23.1) | |
| **Prognostic group by MSKCC class** | | | | 0.362 | | | 0.003 |
| I | 24 (19.4) | 8 (17.0) | 4 (36.4) | | 1 (5.0) | 3 (11.5) | |
| II | 27 (21.8) | 17 (36.2) | 3 (27.3) | | 5 (25.0) | 2 (7.7) | |
| III | 73 (58.9) | 22 (46.8) | 4 (36.4) | | 14 (70.0) | 21 (80.1) | |
| **Loss follow up** | 6 (4.8) | | | | | | |
| **Treatment related mortality** | 9/104 (8.7) | 2 (4.3) | 4 (36.4) | 0.006 | 3 (15.0) | 0 | <0.001 |

Data are median (range), n (%), or n/N (%). ECOG: Eastern Cooperative Oncology Group; IELSG: International Extranodal Lymphoma Study Group; MSKCC: Memorial Sloan-Kettering Cancer Center; ID-MTX: intermediate-dose methotrexate; RT: radiotherapy

**Supplementary Figure 1.** Outcomes of patients who underwent Protocol S with or without Rituximab. (A) Overall survival (B) Progression-free survival

(A)

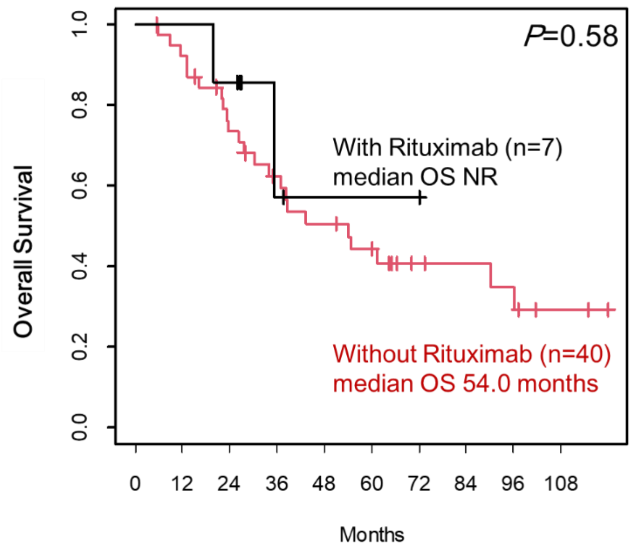


(B)

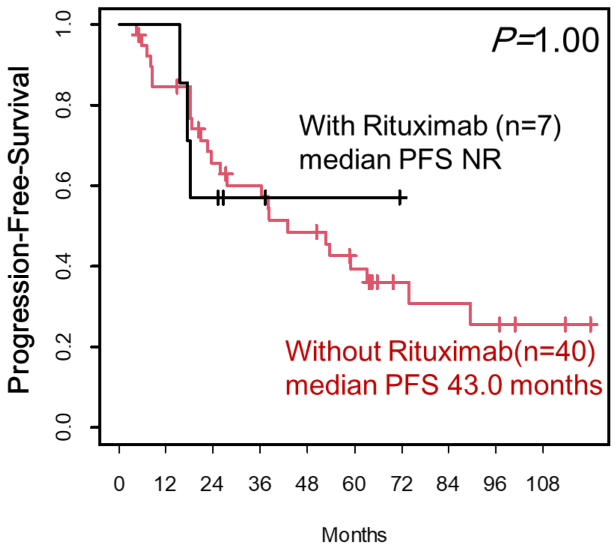

Supplement: Supplementary file 1 — Supplementary file1 (PDF 411 KB) [file 10238_2023_1182_MOESM1_ESM.pdf]
